# Supplementary material for: Empirical methods for controlling false positives and estimating confidence in ChIP-Seq peaks
Source: BMC Bioinformatics. 2008 Dec 5;9:523. doi: 10.1186/1471-2105-9-523 (PMC2628906; doi:10.1186/1471-2105-9-523)
Supplement: Additional File 1 — A variety of html documents from the USeq web site detailing the available applications, their best usage, output file type descriptions, command line menus, etc. [file 1471-2105-9-523-S1.zip › USeqUserGuides/JavaDoc.zip!/JavaDoc/util/bio/xamer/Xamer.html]

Xamer


|  |  |  |
| --- | --- | --- |
| **Overview** \| **Package** \| **Class** \| **Use** \| **Tree** \| **Deprecated** \| **Index** \| **Help** | |  |
| PREV CLASS   **NEXT CLASS** | **FRAMES**    **NO FRAMES**     **All Classes** |  |
| SUMMARY: NESTED \| FIELD \| CONSTR \| METHOD | DETAIL: FIELD \| CONSTR \| METHOD |  |


---


## util.bio.xamer Class Xamer

```
java.lang.Object
  util.bio.xamer.Xamer
```

---

``` public class Xamer extends java.lang.Object ```

Xamer counting related to SELEX.

---

**Constructor Summary**

`Xamer()`


| **Method Summary** | |
| --- | --- |
| `void` | `calculateHitsPerRound()` |
| `void` | `calculatePercentHitsPerRound(int[] totals)` |
| `int[]` | `getHitsPerRound()` |
| `Xamer` | `getRevCompRef()` |
| `java.util.ArrayList` | `getSelexOligos()` |
| `java.lang.String` | `getXamer()` |
| `boolean` | `isPrintMe()` |
| `void` | `setPrintMe(boolean b)` |
| `void` | `setRevCompRef(Xamer xamer)` |
| `void` | `setSelexOligos(java.util.ArrayList list)` |
| `void` | `setXamer(java.lang.String string)` |
| `java.lang.String` | `toStringBarGraph(int[] totals)`             Run pairwise comparisons between percentHitsPerRound if any round is less than the others return true public boolean isEnriched(){ int tester =0; int len = percentHitsPerRound.length; for (int i=0; i |
| `java.lang.String` | `toStringPercent(int[] totals)` |

**Methods inherited from class java.lang.Object**

`equals, getClass, hashCode, notify, notifyAll, toString, wait, wait, wait`

**Constructor Detail**

### Xamer

```
public Xamer()
```


**Method Detail**

### toStringBarGraph

```
public java.lang.String toStringBarGraph(int[] totals)
```

:   Run pairwise comparisons between percentHitsPerRound
    if any round is less than the others return true
    public boolean isEnriched(){
    int tester =0;
    int len = percentHitsPerRound.length;
    for (int i=0; i

---


### toStringPercent

```
public java.lang.String toStringPercent(int[] totals)
```

---


### calculatePercentHitsPerRound

```
public void calculatePercentHitsPerRound(int[] totals)
```

---


### calculateHitsPerRound

```
public void calculateHitsPerRound()
```

---


### getHitsPerRound

```
public int[] getHitsPerRound()
```

---


### getSelexOligos

```
public java.util.ArrayList getSelexOligos()
```

---


### setSelexOligos

```
public void setSelexOligos(java.util.ArrayList list)
```

---


### getXamer

```
public java.lang.String getXamer()
```

---


### setXamer

```
public void setXamer(java.lang.String string)
```

---


### getRevCompRef

```
public Xamer getRevCompRef()
```

---


### setRevCompRef

```
public void setRevCompRef(Xamer xamer)
```

---


### isPrintMe

```
public boolean isPrintMe()
```

---


### setPrintMe

```
public void setPrintMe(boolean b)
```


---


|  |  |  |
| --- | --- | --- |
| **Overview** \| **Package** \| **Class** \| **Use** \| **Tree** \| **Deprecated** \| **Index** \| **Help** | |  |
| PREV CLASS   **NEXT CLASS** | **FRAMES**    **NO FRAMES**     **All Classes** |  |
| SUMMARY: NESTED \| FIELD \| CONSTR \| METHOD | DETAIL: FIELD \| CONSTR \| METHOD |  |


---
